# Quantitative MR Perfusion for the Differentiation of Recurrence and Radionecrosis in Hypoperfusion and Hyperperfusion Brain Metastases After Gamma Knife Radiosurgery

**DOI:** 10.3389/fneur.2022.823731

**Published:** 2022-03-18

**Authors:** Yang Yunqi, Niu Aihua, Zheng Zhiming, Liu Yingchao, Wang Qiang, Ming Yang, Zhang Yi

**Affiliations:** ^1^Department of Neurosurgery, Dongming People's Hospital, Heze, China; ^2^Department of Neurosurgery, Shandong Provincial Hospital Affiliated to Shandong First Medical University, Jinan, China; ^3^Department of Human Resources, Shandong Provincial Hospital Affiliated to Shandong First Medical University, Jinan, China; ^4^Department of Neurosurgery, The Affiliated Hospital of Southwest Medical University, Luzhou, China; ^5^Department of Radiology, Shandong Provincial Hospital Affiliated to Shandong First Medical University, Jinan, China

**Keywords:** brain metastases (BMs), MR perfusion, radionecrosis (RN), gamma knife radiosurgery (GKRS), recurrence

## Abstract

**Objectives:**

Dynamic susceptibility contrast perfusion weighted imaging (DSC-PWI) plays an important role in the differential diagnosis between radionecrosis and recurrence of brain metastases (BMs) after gamma knife radiosurgery (GKRS). While the perfusion condition of preliminary hyperperfusion and hypoperfusion BMs when recur has not been studied, as well the separating performance of quantitative DSC-PWI in both kinds of BMs.

**Methods:**

From February 2017 to October 2019, quantitative DSC-PWI was performed in patients with untreated BMs in this observational study. Patients were assigned to hyperperfusion and hypoperfusion group according the quantitative cerebral blood volume (qCBV). During follow-up after GKRS, patients with a diagnostic pitfall of radionecrosis and recurrence accepted second quantitative DSC-PWI. Final diagnosis was based on the histological results or follow-up results. Receiver operating curve analysis was used to explore the performance of qCBV.

**Results:**

Twenty-nine patients (mean age: 61.3 ± 9.4 years old; male/female: 13/16) were assigned to the group of hypoperfusion group, and 26 patients (mean age: 58 ± 10.4 years old; male/female: 14/12) to hyperperfusion group. The mean qCBV values between hypoperfusion and hyperperfusion groups when recurred were not significantly different (3.17 ± 0.53 ml/100 g vs. 3.27 ± 0.47 ml/100 g, *p* = 0.63). qCBV was feasible to separate radionecrosis and recurrence in both groups (AUC=0.94 and AUC=0.93, separately).

**Conclusion:**

Both premilitary hyperperfusion and hypoperfusion BMs would transform to a high microvascular density when recurs. qCBV is feasible to distinguish radionecrosis and recurrence among both kinds of BMs after GKRS.

## Introduction

Brain metastases (BMs) are among the most common tumors in the central nervous system, with a poor prognosis and high morbidity and mortality (1). Radiation therapy is an alternative for subsequent treatment. In recent years, gamma knife radiosurgery (GKRS) has become an increasingly popular choice for patients diagnosed with BM because it leads to only limited treatment-induced morbidity (2). Even so, radionecrosis can still occur among some patients after GKRS (3), which is difficult to separate from recurrence using conventional structural images.

Dynamic susceptibility contrast perfusion weighted imaging (DSC-PWI) is one of the most commonly used methods in the differential diagnosis of radionecrosis and tumor recurrence for both gliomas and BMs (4–6). The popular interpretation is that tumor recurrence is characterized by increased angiogenesis, but radionecrosis is not (4, 6, 7). However, in clinical practice, doubts persist. High-grade gliomas are accompanied by obvious angiogenesis; therefore, recurrent high-grade gliomas show an obviously increased cerebral blood volume (CBV) in the irradiated area. However, as the two most common types (8), brain metastases originating from lung and breast tumors are considered hypoperfusion tumors, which are different from high-grade gliomas (9, 10). Theoretically, recurrent BMs would not show increased CBV because these BMs are hypoperfused before GKRS treatment.

Previous studies did not separate recurrent BMs into hyperperfusion and hypoperfusion BMs before analysis (5, 9–12). In other words, they did not report the untreated perfusion conditions of recurrent BMs. Therefore, it is still unknown whether hypoperfusion BMs transform to hyperperfusion BMs when they recur. The technical limitations of conventional DSC-PWI may be a response to this uncertain condition because rCBV is a qualitative parameter, which is not qualified enough for comparisons across different scanning times (13). Quantitative DSC-PWI is urgent for this work. Currently, the Bookend DSC-PWI technique, which uses pre- and postcontrast T1 maps to calibrate a conventional DSC sequence, is a quantitative DSC-PWI technique that enables comparisons across different scanning times (14, 15).

With the help of quantitative CBV (qCBV) derived from Bookend DSC-PWI, this study aimed to investigate the perfusion condition of hyperperfusion and hypoperfusion BMs when they recur, as well the separation performance of qCBV in both kinds of BMs.

## Materials and Methods

### Study Population

From February 2017 to October 2019, 340 patients with BMs were treated by GKRS in Shandong Provincial Hospital Affiliated to Shandong First Medical University. Patients who met the inclusion criteria were enrolled in this retrospective study. The inclusion criteria were as follows: [1] histopathological diagnosis of primary cancer; [2] target BM lesions that met the criterion of Response Assessment in Neuro-Oncology Brain Metastases (RANO-BM); [3] treatment by GKRS; [4] newly enhanced lesions or enlarged enhanced lesions were revealed inside the irradiated nidus after injection of contrast agent during follow-up MRI examination; and [5] postirradiation period > 5 months. Among the 340 patients, 96 met the inclusion criteria.

However, 41 patients were excluded for the following reasons: [1] no qCBV map before GKRS (*n* = 16); (2) no qCBV map when diagnosed with recurrence or radionecrosis (*n* = 11); [3] unqualified MRI data (e.g., severe head motions, *n* = 5); [4] no follow-up MRI data or pathological results (*n* = 9). Finally, 55 patients (age: 59.8 ± 9.9 years old, male/female: 27/28) were enrolled in this retrospective study.

Written informed consent was obtained from all patients before commencement of the study and after receiving approval from the hospital's ethical committee. All experiments were performed in compliance with the Declaration of Helsinki.

### MRI Protocol

All patients underwent MR scans on a MAGNETOM 3T scanner (Skyra, Siemens, Erlangen, Germany) using a 20-channel head-neck coil. The MR exam included a routine protocol for brain examination (T2WI, precontrast T1WI, and T2-FLAIR) and a prototype Bookend DSC-PWI sequence called ScalePWI. ScalePWI could be acquired only after the approval of Siemens Healthineers. The ScalePWI sequence merged the pre- and postcontrast T1 mapping into the GRE-EPI sequence for DSC-PWI. The details of the descriptions of ScalePWI were described previously (16). The imaging parameters of ScalePWI were as follows: TR/TE 1,600 ms/30 ms, bandwidth 1,748 Hz/pixel, 21 axial slices, field of view (FOV) 220 × 220 mm, voxel size 1.8 × 1.8 × 4 mm^3^, slice thickness 4.0 mm, and a flip angle (FA) of 90 degrees. For each slice, 50 measurements were acquired for DSC-PWI analysis. After 46 s of injector delay, 0.2 mmol per kg bodyweight of contrast agent (Gd-DTPA, Magnevist; Schering, Berlin, Germany) was administered, followed by a 20-ml saline flush. An injection velocity of 4.0 ml/s was introduced in this study. Quantification of cerebral blood volume is based on the Bookend technique, where the value of qCBV is dependent on the change of white matter before and after the injection of contrast agent:$CBV_{WM}=\frac{{\left(\frac{1}{T_{1}^{Post}}-\frac{1}{T_{1}^{Pre}}\right)}_{WM}}{{\left(\frac{1}{T_{1}^{Post}}-\frac{1}{T_{1}^{Pre}}\right)}_{Blood}}\times 100\%$,quantification of CBV_WM_: $qCBV_{WM}=WCF\left(R_{1}\right)\times \frac{1}{\rho }\times \frac{1-Hct_{LV}}{1-Hct_{SV}}\times CBV_{WM}$, where$WCF\;\left(R_{1}\right)=8.2\times 10^{-3}R_{1}^{2}+0.25R_{1}+0.51$,ρ*Hct*_*LV*_*Hct*_*SV*_*R*_1_ were all constant values. The qCBV of each voxel was calculated by the following functions: $qCBV=rCBV\times \frac{qCBV_{WM}}{rCBV_{WM}}$. Unit of qCBV is ml/100 g.

Then, a postcontrast T1-weighted sequence with the same slices as ScalePWI was performed. The imaging parameters were as follows: TR/TE 250/2.5 ms, TI 900 ms, FOV 220 × 220 mm, slice thickness 4 mm, and FA 70 degrees. The imaging protocol was the same for all patients.

### Tumor Segmentation

Target lesions were chosen based on the principle of RANO-BM (17), but only the largest lesion was chosen for one patient. For both untreated BM lesions and follow-up BM lesions, regions of interest (ROIs) were drawn completely by hand to only cover the enhancing area inside the lesions on postcontrast T1W images, avoiding major cortical vessels adjacent to the lesions. The ROIs were copied to the qCBV map, and the mean value of ROIs represented the qCBV of lesions. These works were performed via ITK-SNAP (version 3.8.0, www.itksnap.org) by two neuroradiologists together (with over 10 and 15 years of experience, each), who were blind to the diagnostic results.

### Group Assignment of the BMs Before GKRS

A previous study confirmed that a qCBV value of 2.0 ml/100 g was a good cut-off value for distinguishing tumor recurrence and radionecrosis of BM after GKRS (18). In addition, the gray matter in the basal ganglia region was ~2.0 ml/100 g (19). Therefore, we chose this value as the cut-off for separating hyperperfusion lesions and hypoperfusion lesions among untreated BMs. Lesions with a mean qCBV value ≤2.0 ml/100 g were considered hypoperfusion BMs, while those over 2.0 ml/100 g were considered hyperperfusion BMs.

### Diagnosis of Newly or Enlarged Enhanced Lesions

Although histopathological confirmation is the gold standard for differentiating radionecrosis and tumor recurrence of BMs after GKRS, it can be obtained only in a small group of patients. Radiographic and clinical assessments are the most commonly used identification methods (17). Patients' conditions and lesions were thereafter evaluated based on clinical manifestations and routine MR imaging every 3 months. We made the final diagnosis of radiation necrosis when the target lesion showed complete response, partial response, or stable disease depending on the RANO-BM method in subsequent follow-up MR images after a minimum of 6 months (median 12.5 months, range: 6–21 months). If the lesion presented with progression on serial MR examination and the patient's neurological condition deteriorated progressively, we diagnosed the case as tumor recurrence. Clinical assessments were performed by a neurosurgeon (with 12 years of experience), and radiographic assessments were performed by a neuroradiologist (with 18 years of experience). Divergence between clinical and radiographic assessments was resolved by stereotactic biopsy or craniotomy. In addition, the change in qCBV (ΔqCBV) between untreated qCBV (*qCBV*_*pre*_) and posttreated qCBV (*qCBV*_*post*_) was also calculated, which is defined as Δ*qCBV* = *qCBV*_*post*_−*qCBV*_*pre*_.

### Statistical Analysis

The Kolmogorov–Smirnov test was used to check for the normal distribution of data. Differences in normally distributed data were tested using an unpaired *t* test; non-normally distributed variables were tested using the Mann–Whitney U test. Differences in count variables were tested using the chi-squared test. The difference in qCBV between untreated and recurrent lesions was tested using a paired *t* test. The DeLong test was performed to evaluate the difference in the ROCs. A two-sided *p*-value < 0.05 was considered significantly different. All calculations were performed using SPSS (Statistics version 20, IBM, Armonk, NY).

## Results

### Study Population

Among the 55 enrolled patients, 29 patients (mean age: 61.3 ± 9.4 years old; male/female: 13/16) were assigned to the group of hypoperfusion BMs, and 26 patients (mean age: 58 ± 10.4 years old; male/female: 14/12) were assigned to the group of hyperperfusion BMs. In addition, 15 and 14 cases were diagnosed as recurrence and radionecrosis in the hypoperfusion BM group, respectively. Fourteen and 12 patients were diagnosed with recurrence and radionecrosis, respectively, in the hypoperfusion BM group. Finally, eight patients (8/29, 27.6%) with recurrence and four patients with radionecrosis (4/26, 15.5%) were diagnosed by stereotactic biopsy or craniotomy. The patients were diagnosed by the depending on the RANO-BM method in subsequent follow-up MR images after a minimum of 6 months. Detailed information on the entire enrolled population is summarized in Table 1. The underlying histological subtypes of the primary tumors are summarized in Table 2.

**Table 1:** The summarized information of all enrolled populations.

	**Hyperperfusion group**	**Hypoperfusion group**	***P* value**
Age, years	58 ± 10.4	61.3 ± 9.4	0.23
Gender, male/female	14/12	13/16	0.59
Primary tumor, n			0.006
Lung	8	18	
Breast	4	8	
Kidney	9	0	
Digestive tract	2	2	
Melanoma	3	1	
Locations, n			0.94
Frontal	6	7	
Parietal	8	7	
Temporal	3	5	
Occipital	3	4	
Cerebellum	5	4	
Brainstem	1	2	
KPS, median [IQR]	70 [70.90]	70 [70,90]	0.63
MD, median [IQR]	2.3 [1.7, 2.5]	2.5 [1.7, 3.2]	0.27
Dose, median [IQR]	18 [18, 21]	18 [18, 21]	0.71

*KPS, Karnofsky performance status; IQR, interquartile range; MD, maximum diameter*.

**Table 2:** The detailed results of comparisons between two groups.

**Primary tumor**	**Histological subtype**	**Hyperperfusion group**	**Hypoperfusion group**
Lung	Squamous carcinoma	3	7
	Adenocarcinoma	2	9
	small cell lung cancer	3	2
Breast	Invasive ductal carcinoma	2	4
	Invasive lobular carcinoma	2	2
	Adenocarcinoma	0	2
Kidney	Renal clear cell carcinoma	7	0
	Papillary renal cell carcinoma	2	0
Digestive tract	Adenocarcinoma	2	2
Melanoma	Superficial spreading melanoma	3	1

### Comparisons Between Hyperperfusion and Hypoperfusion Groups

The mean qCBV between untreated and recurrent lesions in the hypoperfusion group was significantly different (1.40 ± 0.32 ml/100 g vs. 3.0 ± 0.58 ml/100 g, *p* < 0.001), and that in the hyperperfusion group was not (3.18 ± 0.68 ml/100 g vs 2.89 ± 0.58 ml/100 g, *p* = 0.254). The mean qCBV between the hyperperfusion and hypoperfusion groups was not significantly different with recurrence (*p* = 0.62) or radionecrosis (*p* = 0.45). In addition, the mean ΔqCBV between the two groups was significantly different with recurrence and radionecrosis (both *p* < 0.001). The detailed results of comparisons between the two groups are summarized in Table 3.

**Table 3:** The detailed results of comparisons between two groups.

	**Hyperperfusion group**	**Hypoperfusion group**	***P* value**
Recurrence	2.89 ± 0.58	3.0 ± 0.58	0.62
Radionecrosis	1.59 ± 0.31	1.68 ± 0.26	0.45
ΔqCBV_recurrence_	0.15 [−0.3, 0.2]	2.0 [1.6, 2.3]	<0.001
ΔqCBV_radionecrosis_	−1.92 ±−0.68	0.18 ± 0.39	<0.001

### Diagnostic Performance in Two Groups and All Patients

**Figure 1:**
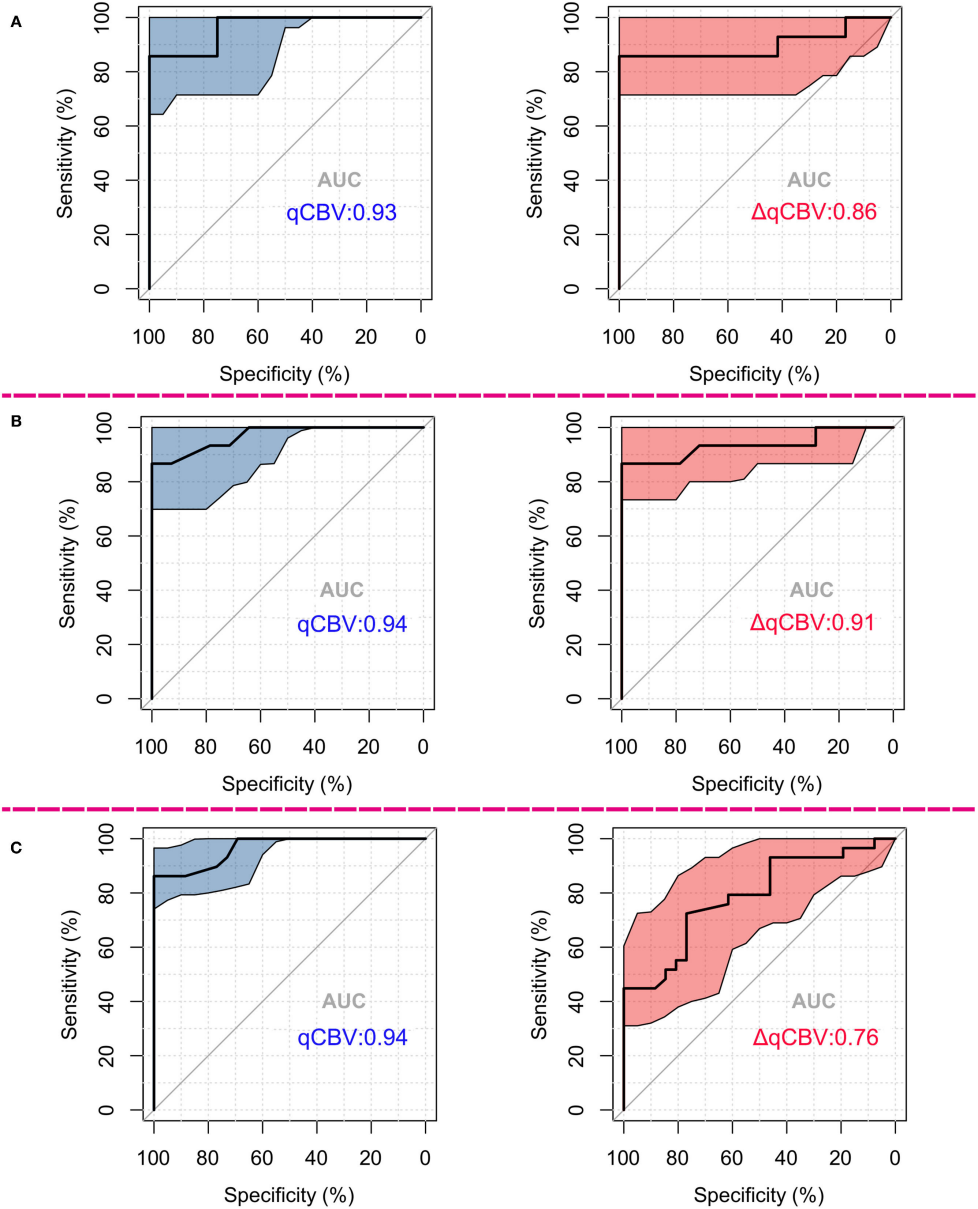
ROCs differentiating radionecrosis from recurrence of BMs after GKRS treatment. **(A)** ROCs in hyperperfusion groups; **(B)** ROCs in hypoperfusion groups; **(C)** ROCs among all mixed patients. For **(A–C)**, the left subfigure shows the ROCs of qCBV, and the right subfigure shows those of ΔqCBV. The colored area in the map indicates the 95th percentile confidence interval of the AUC. ROC, receiver operating curve; GKRS, gamma knife radiosurgery; AUC, area under the ROC.

**Figure 2:**
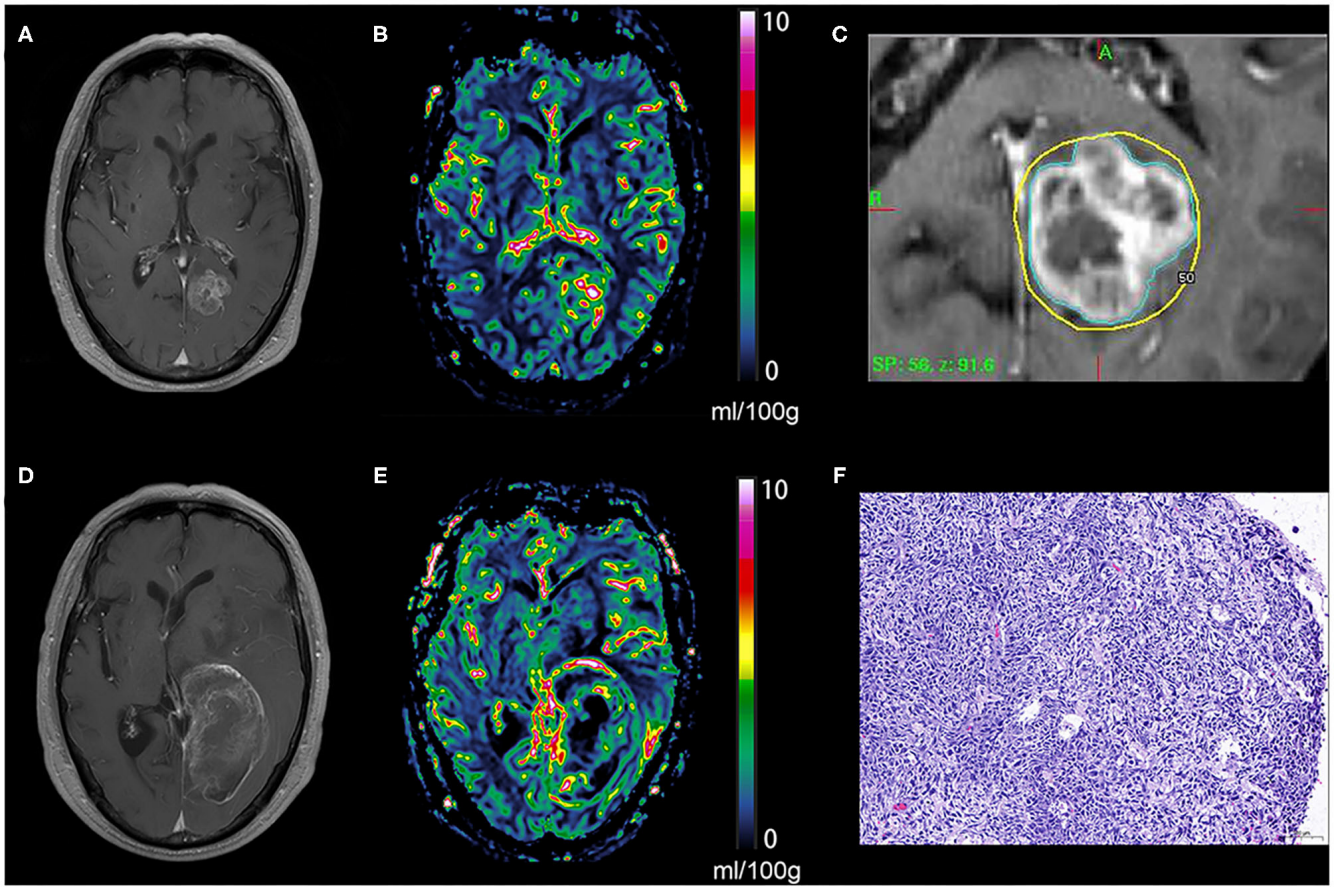
Tumor recurrence of the hyperperfusion BM. An enhancing lesion was discovered in the left occipital lobe of a 69-year-old patient with lung cancer **(A)**, a quantitative CBV map revealed that the lesion was hyperperfused **(B)**, and gamma knife radiosurgery was performed later **(C)**. One year later, the enhancing lesion was enlarged **(D)**, and a quantitative CBV map revealed that the enlarged lesion was still hyperperfused **(E)**. The lesion was removed via surgical operation, and the pathological result was brain metastasis recurrence **(F)**. BM, brain metastases; CBV, cerebral blood volume.

**Figure 3:**
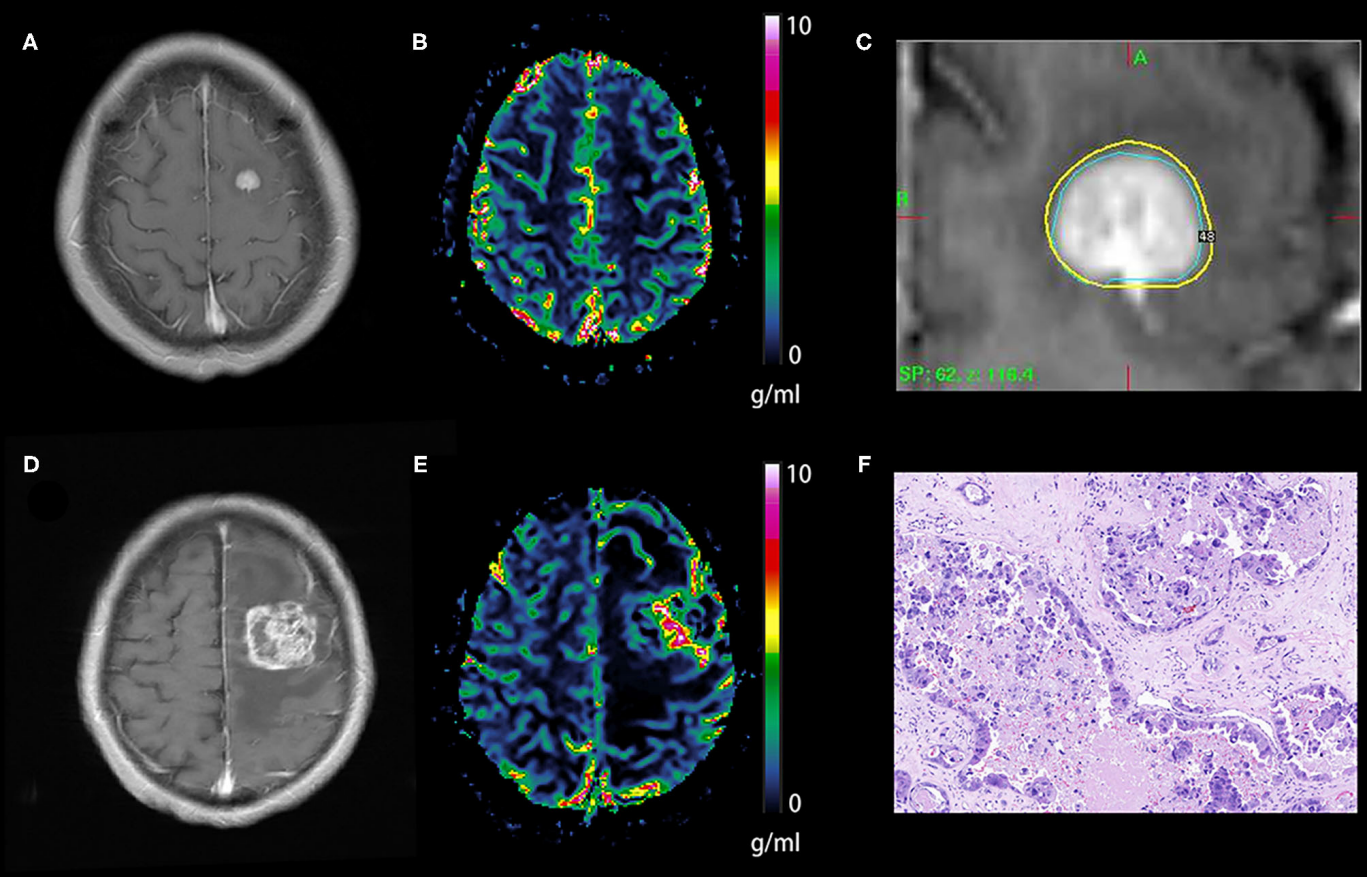
Tumor recurrence of the hypoperfusion BM. An enhancing lesion was discovered in the left frontal lobe of a 50-year-old patient with breast cancer **(A)**. A quantitative CBV map revealed that the lesion was hypoperfused **(B)**, and gamma knife radiosurgery was performed later **(C)**. Ten months later, the enhancing lesion was enlarged **(D)**, and a quantitative CBV map revealed that the enlarged lesion transformed to hyperperfusion **(E)**. The lesion was removed via surgical operation, and the pathological result was brain metastasis recurrence **(F)**. BM, brain metastases; CBV, cerebral blood volume.

The diagnostic performance of qCBV in the two groups and all patients was explored first. qCBV had a similar diagnostic performance in the two groups and all patients. In addition, ΔqCBV performed worse than qCBV. Furthermore, ΔqCBV in all patients performed worst. The ROC curves of qCBV and ΔqCBV under the three conditions are summarized in Figure 1. Detailed information on the ROC analysis is summarized in Table 4. Examples of hyperperfusion BMs and hypoperfusion BMs are shown in Figures 2, 3.

**Table 4:** The detailed information of ROC analysis.

	**AUC**	**Accuracy**	**Specificity**	**Sensitivity**	**PPV**	**NPV**
**qCBV**
Hyperperfusion	0.93	0.89	0.97	0.83	0.97	0.83
Hypoperfusion	0.94	0.90	0.96	0.84	0.97	0.85
All	0.94	0.90	0.97	0.83	0.97	0.84
**ΔqCBV**
Hyperperfusion	0.86	0.89	0.97	0.83	0.97	0.83
Hypoperfusion	0.91	0.90	0.97	0.84	0.97	0.84
All	0.76	0.72	0.74	0.69	0.75	0.68

## Discussion

DSC-PWI plays an important role in the differential diagnosis between radionecrosis and tumor recurrence of BMs after GKRS treatment. However, the performance of quantitative DSC-PWI in hyperperfusion and hypoperfusion BMs has not been investigated separately. Our results demonstrated that quantitative DSC-PWI was feasible to separate radionecrosis from recurrence of BMs after GKRS treatment for both kinds of BMs. In addition, both kinds of BMs appear as hyperperfusion when recurring.

Unlike high-grade gliomas, BMs are not always hyperperfused. Traditionally, BMs from lung and breast cancer, which are the most common causes of BMs, have been treated as hypoperfusion lesions (9). Those from kidney cancer and melanoma are treated as hyperperfusion lesions (8). In this study, however, some BMs from lung and breast cancer presented as hyperperfusion lesions, while BMs from melanoma can also present as hypoperfusion lesions. In addition, all of the BMs from kidney cancer presented with hyperperfusion, which is consistent with a previous study (5, 10). The results may reveal heterogeneous perfusion conditions in BMs. We observed that most of [23 of 26] these hyperperfusion lesions were solid or solid-cystic, while most [24 of 29] hypoperfusion lesions were cystic or unsolid. These results may indicate that the solid part of BMs could result in high overexpression of VEGF (13). Therefore, according to our findings, evaluation of the perfusion conditions of untreated BMs referring only to the origination of the tumor is not correct. MR perfusion should be performed when evaluating the perfusion conditions of untreated BMs.

The feasibility of DSC-PWI in distinguishing radionecrosis from recurrence of BMs after radiotherapy has been validated by many previous studies (5, 9–11). Few studies have reported the performance of quantitative DSC-PWI [18]. In addition, no studies have explored the performance of quantitative DSC-PWI in hyperperfusion and hypoperfusion BMs separately. Our results showed that quantitative DSC-PWI had good separation performance in both hyperperfusion and hypoperfusion BMs, as well as in a mixed population. Interestingly, for the preliminary hypoperfusion BMs, most of them showed a greatly improved qCBV value when they recurred. This means that the hypoperfusion BMs would transform to hyperperfusion BMs when they recur. The reasons for the change in perfusion conditions in hypoperfusion BMs may be that radiotherapy induced VEGF overexpression in tumor cells or that radiotherapy-resistant tumor cells transformed to higher malignancy with VEGF overexpression when they recurred.

Due to the limitation of artery input functions (AIFs) of DSC-PWI, it could be a challenge to use this technology as a quantitative approach to evaluate the perfusion conditions of brain tumors. Bookend DSC-PWI provided an AIF-independent approach to solve this problem (15). With the help of the Bookend technique, quantitative CBV is feasible in the longitudinal and transversal assessment of perfusion conditions of BMs. Our results demonstrated that the difference (ΔqCBV) between untreated qCBV and posttreated qCBV at diagnosis did not perform better than qCBV in separating radionecrosis from tumor recurrence. Moreover, ΔqCBV is not applicable in a mixed population because it had the worst performance under this condition. The reason may be that the preliminary hyperperfusion BMs would still be hyperperfusion BMs when diagnosed as recurrence, and the preliminary hypoperfusion BMs would still be hypoperfusion BMs when diagnosed as radionecrosis, and a large overlap would be inevitable under both kinds of conditions.

Some limitations should be addressed here. First, the size of the study population was relatively small, and further studies with larger populations should be performed to validate our findings. Second, only a limited number of patients were validated by pathological results because only a small portion of BMs need neurosurgical intervention after GKRS. In addition, for patients with a probability of radionecrosis, this is an inherent dilemma in clinical treatment because the risk of possible complications of biopsy might outweigh the benefits of histopathological diagnosis (1).

In conclusion, hypoperfusion BMs transform to hyperperfusion BMs when they recur. Quantitative CBV could still be feasible to distinguish radionecrosis from tumor recurrence in both hyperperfusion and hypoperfusion BMs after GKRS treatment. Further studies with larger and multicentre cohort validation should be performed to validate our findings.

## Data Availability Statement

The raw data supporting the conclusions of this article will be made available by the authors, without undue reservation.

## Ethics Statement

The studies involving human participants were reviewed and approved by Shandong Provincial Hospital Affiliated to Shandong First Medical University. The patients/participants provided their written informed consent to participate in this study.

## Author Contributions

MY and ZY: guarantor of integrity of the entire study. YY and NA: study concepts, design, and manuscript preparation. WQ and LY: literature research and clinical studies. YY and MY: experimental studies/data analysis. NA and ZY: statistical analysis. YY, NA, ZZ, LY, WQ, MY, and ZY: manuscript editing. All authors contributed to the article and approved the submitted version.

## Conflict of Interest

The authors declare that the research was conducted in the absence of any commercial or financial relationships that could be construed as a potential conflict of interest.

## Publisher's Note

All claims expressed in this article are solely those of the authors and do not necessarily represent those of their affiliated organizations, or those of the publisher, the editors and the reviewers. Any product that may be evaluated in this article, or claim that may be made by its manufacturer, is not guaranteed or endorsed by the publisher.
